# Construction and validation of a risk-prediction model for hypovolemic shock after cytoreductive surgery in patients with ovarian cancer: a retrospective study

**DOI:** 10.7717/peerj.20976

**Published:** 2026-03-20

**Authors:** Cong An, Yingyan Yao, Dong Weng, Hang Hu, Jing Jiang, Mingou Cui

**Affiliations:** 1 Intensive Care Unit, Zhejiang Cancer Hospital, Hangzhou, China; 2 Hepatobiliary and Pancreatic Surgery, Zhejiang Cancer Hospital, Hangzhou, China

**Keywords:** Hypovolemic shock, Ovarian cancer, Prediction model, Risk factors, Cytoreductive surgery

## Abstract

**Introduction:**

Ovarian cancer patients undergoing cytoreductive surgery are prone to hypovolemic shock in the early postoperative period, resulting in tissue hypoperfusion, lactic acid accumulation, endotoxin displacement, and even multiple organ dysfunction syndrome; however, in existing studies, there is a lack of a dynamic approach to assess the risk of postoperative hypovolemic shock. This study aimed to construct and validate a visual prediction model of hypovolemic shock after cytoreductive surgery.

**Methods:**

This is a retrospective observational study. Patients with ovarian cancer who received cytoreductive surgery at Zhejiang Cancer Hospital between January 2023 and June 2024 were retrospectively enrolled and divided into a training group and a validation group. Independent predictors of hypovolemic shock were identified using least absolute shrinkage and selection operator (LASSO) regression from the training set, and a nomogram was constructed based on these predictors. A nomogram was used to depict the weight of each variable in the logistic regression model on the event occurrence. The performance of the nomogram was assessed using receiver operating characteristic (ROC), calibration curve, and decision curve analysis (DCA).

**Results:**

A total of 423 patients were eligible for inclusion in this study. There were 301 cases in the training group and 122 cases in the validation group. This visual prediction model was constructed based on the duration of the operation (odds ratio (OR) = 1.273; 95% confidence interval (CI) [1.052–1.552]), the amount of blood lost during the operation (OR = 1.102; 95% CI [1.020–1.196]), the amount of albumin (OR = 0.935; 95% CI [0.879–0.993]) and fibrinogen (OR = 0.606; 95% CI [0.371–0.948]) immediately after the operation, and the postoperative use of sedative drugs (OR = 2.248; 95% CI [1.109–4.538]). The area under the ROC of the nomogram for the training and validation cohorts was 0.800 (95% CI [0.740–0.860]) and 0.821 (95% CI [0.735–0.907]), respectively. The predicted probabilities of the two groups of models were basically consistent with the actual incidence rates, with the average absolute errors being 0.016 and 0.019, respectively. The Hosmer-Lemeshow test of the training group (*P* = 0.722) and the validation group (*P* = 0.565) showed that there was no significant difference between the predicted values and the actual values, indicating that the model fitted well. The results of the DCA curve showed that the two groups had a net benefit within the probability range of risk threshold values of 0.01 to 0.84 and 0.04 to 0.80, respectively.

**Conclusions:**

The model constructed in this study demonstrates improved predictive accuracy for the risk of hypovolemic shock after cytoreductive surgery in patients with ovarian cancer, and it holds the potential to provide a basis for medical staff to achieve early identification and timely intervention.

## Introduction

According to the International Agency for Research on Cancer, there were 324,603 new cases of ovarian cancer worldwide in 2022 (International Agency for Research on Cancer, 2022). At present, the mainstay of treatment modalities for ovarian cancer are surgery and chemotherapy (Climent et al., 2023; De Bree, Michelakis & Anagnostopoulou, 2022; Finch & Chi, 2023), with cytoreductive surgery being the cornerstone of treatment strategy to significantly improve the survival rates and prognosis of patients (Chen, 2024). A study showed that more than 60% of ovarian cancer patients had already developed extra-pelvic metastasis at the time of diagnosis (National Cancer Institute, 2024). Due to the characteristics of invasive tumor growth, satisfactory cytoreductive surgery involves multiple organs, a wide range of surgical resections and a prolonged operation time (Orr, 2018; Fotopoulou et al., 2021; Schmeler et al., 2010). These factors increase the risk of hypovolemic shock in the early postoperative period. Raising evidence suggests that hypovolemia with hypoperfusion contributes to the occurrence of serious postoperative complications (Egger et al., 2022; Grass et al., 2020). If the hypovolemic shock is not recognized and prevented in time, it will result in reduced effective circulating blood volume, tissue hypoperfusion, cell metabolism disorder and multiple organ dysfunction syndrome (Kalkwarf & Cotton, 2017; Ledgerwood & Blaisdell, 2012). Nevertheless, current studies focus on other postoperative complications of ovarian cancer, such as postoperative bleeding, anastomotic fistula, surgical site infection and perforation (Narasimhulu et al., 2020; Bernard & Boucher, 2020; Navarro et al., 2023). Although goal-directed hemodynamic and fluid management strategies have been developed to prevent postoperative hypovolemic shock (Egger et al., 2024; Russo et al., 2018), their scope is predominantly limited to intraoperative volume management, which fails to identify specific postoperative risks due to the absence of dedicated assessment and screening tools, thus rendering the effective prevention of this condition a persistent challenge.

Following this, we performed a retrospective analysis of patients who underwent cytoreductive surgery for ovarian cancer in our hospital. Ultimately, a nomogram prediction model was established, aiming to provide a basis for clinical staff to accurately predict the risk of hypovolemia after ovarian cancer surgery and formulate intervention measures.

## Materials & Methods

### Study design and population

This retrospective study was conducted in Zhejiang Cancer Hospital and has been approved by the Medical Ethics Committee of Zhejiang Cancer Hospital, approval number: IRB-2024-307(IIT). The study used information data generated from clinical treatment, and all patients signed informed consent upon admission, agreeing to authorize their clinical diagnosis and treatment data for this research. Data were collected from patients with ovarian cancer who were treated in the intensive care unit from January 2023 to June 2024. Inclusion criteria were as follows: (1) patients with a diagnosis of ovarian cancer; (2) patients at least 18 years of age; and (3) patients who received cytoreductive surgery. Exclusion criteria: patients with incomplete electronic medical record systems and lacking the data and information required for this study. A total of 423 patients were included in the study, including 301 in the training group and 122 in the validation group.

### Diagnostic criteria and definitions

Diagnosis of hypovolemic shock was made according to diagnostic criteria proposed by the European Society of Critical Care Medicine in 2014: Diagnosis of hypovolemic shock was made according to diagnostic criteria proposed by the European Society of Critical Care Medicine in 2014: (1) Systolic blood pressure <90 mmHg, or mean arterial pressure (MAP) <65 mmHg, or a decrease of ≥40 mmHg from baseline; (2) lactate >2 mmol/L; (3) At least one clinical manifestation of insufficient tissue perfusion occurs: the peripheral window (skin that is cold, clammy and blue, pale or discolored); the renal window (decreased urine output: <0.5 mL/kg/h); the neurologic window (altered mental characterized by obtundation, disorientation and confusion) (Cecconi et al., 2014). We defined the risk of hypovolemic shock as the period from the end of surgery to 48 h after surgery.

### Collected data

Through literature review, discussion among members of the research group and expert consultation, we developed a questionnaire on risk factors of hypovolemic shock after tumor cell reduction in patients with ovarian cancer (File S1). All risk factors were evaluated using quantifiable, convenient, specific, and objective clinical indicators. The questionnaire consists of five sections: (1) Preoperative general information: age, weight, tumor staging, Nutritional Risk Screening 2002; (2) medical history: underlying diseases, number of operations, preoperative chemotherapy frequency; (3) intraoperative information: American Society of Anesthesiologists physical status classification system, operation duration, intraoperative fluid replenishment, intraoperative blood loss, intraoperative ascites loss or not, intraoperative blood transfusion; (4) immediate postoperative laboratory parameters: activated partial thromboplastin time (APTT), prothrombin time (PT), albumin, hemoglobin, fibrinogen; (5) postoperative factors: postoperative use of sedative drugs and Postoperative mechanical ventilation.

### Data collection

Before data collection, the members involved should be trained professionally and systematically, and the investigation can only be carried out after passing the assessment. Through the patient electronic medical record system data of Zhejiang Cancer Hospital, the researchers collected multi-dimensional real-world data of patients undergoing ovarian cancer cytoreductive surgery, including preoperative general information, past medical history, conditions during surgery, immediate postoperative laboratory indicators and other postoperative conditions.

### Statistical analysis

The data were input by a double-check method, and the data were processed and analyzed by R language (version 4.3.3; R Core Team, 2024). For normally distributed continuous variables, we described them using the mean ± standard deviation and performed intergroup comparisons with the independent samples *t*-test. When the data was non-normally distributed, we used the median [Q1, Q3] and conducted intergroup comparisons with the rank sum test. Additionally, we presented data as case counts (%), with the chi-square test for intergroup comparisons. If the data did not meet the chi-square test requirements, the Fisher exact probability method was employed. The least absolute shrinkage and selection operator (LASSO) regression, a novel method for variable selection, applies penalized regression to exclude the coefficients of less important variables from the model (Gu et al., 2023). This approach effectively addresses multicollinearity and is particularly useful for handling high-dimensional data and reducing overfitting (Xia et al., 2024). Therefore, based on the results of univariable logistic regression analysis, the LASSO regression algorithm was used to select significant features (coefficients were non-zero). Subsequently, we conducted a multivariate logistic regression analysis (stepwise, bidirectional). Finally, the nomogram of the prediction model was constructed based on variables with *P* < 0.05 in the stepwise method. The bootstrap approach is widely used to assess confidence in species relationships inferred from multiple sequence alignments. It resamples sites randomly with replacement to build alignment replicates of the same size as the original alignment and infers a phylogeny from each replicate dataset (Sharma, 2021). In addition, the calibration curve serves as a visual representation of bootstrap sampling. The calibration curve is used to evaluate whether the probability values predicted by the model are accurate. The Mean Absolute Error is a key indicator that quantifies the average value of the deviation between the predicted probability and the actual observed results. A value less than 0.02 indicates that the model performs excellently in terms of prediction accuracy (Kang et al., 2023). In our study, a total of 1,000 bootstrap samples were sampled to verify the constructed nomogram. Calibration curves were drawn to evaluate the calibration degree of the model, and the Hosmer-Leme-show test was performed to evaluate the goodness of fit. An receiver operator characteristic (ROC) curve is a plot of the sensitivity *versus*—specificity of a diagnostic test, which assesses the model’s ability to correctly distinguish between event participants and non-participants. The area under curve (AUC) is the core quantitative indicator for summarizing the overall discriminative ability represented by the ROC curve (Asha et al., 2023). Therefore, in this study, the recognition efficiency of the model was evaluated by analyzing the ROC curve and the AUC. The decision curve analysis (DCA) provides a framework directly linked to clinical decision-making for evaluating and comparing the clinical practicality of risk prediction models (Van Calster et al., 2018). Hence, a clinical DCA was constructed to evaluate the clinical application value of the model and quantify the net benefit within the threshold probability range. Finally, the constructed model was further verified in the validation group.

## Results

### Patient information and characteristics in training group

There were 301 patients in the training group, among whom 70 patients (23.3%) presented with hypovolemic shock.The univariable logistic regression analysis showed that there were statistically significant differences between the hypovolemic shock group and the non-hypovolemic shock group in operation duration, intraoperative fluid replenishment, intraoperative blood loss, intraoperative blood transfusion, APTT, PT, albumin, hemoglobin, fibrinogen immediately after operation, postoperative use of sedative drugs, and postoperative mechanical ventilation (all *P* < 0.05, Table 1).

**Table 1:** Univariate logistic regression analysis of hypovolemic shock in training group.

Variables	Non-hypovolemic shock group (*n* = 231)	Hypovolemic shock group (*n* = 70)	statistic	*P*
① Age (years)	57.00 (51.00, 63.00)	57.50 (52.00, 65.00)	−0.822	0.411
① Weight (kg)	55.00 (50.00, 62.00)	56.00 (50.62, 62.38)	−0.493	0.622
② Intraoperative ascites loss or not			0.782	0.377
No	177 (76.6)	50 (71.4)		
Yes	54 (23.4)	20 (28.6)		
② Underlying disease			0.925	0.336
No	137 (59.3)	46 (65.7)		
Yes	94 (40.7)	24 (34.3)		
② Tumor staging			6.489	0.090
I	12	7		
II	48	16		
III	59	24		
IV	112	23		
① Preoperative chemotherapy frequency (times)	2.00 (0.00, 3.00)	0.00 (0.00, 3.00)	1.666	0.096
① NRS 2002 (points)	3.00 (2.00, 3.00)	3.00 (2.00, 3.00)	−1.900	0.057
③ ASA				0.235
I	14 (6.1)	4 (5.7)		
II	216 (93.5)	64 (91.4)		
III	1 (0.4)	2 (2.9)		
① Number of operations (times)	1.00 (1.00, 2.00)	1.00 (1.00, 1.00)	1.533	0.125
① Operation duration (hours)	5.50 (4.50, 7.00)	7.25 (5.50, 8.23)	−5.183	<0.001
① Intraoperative fluid replenishment (mL)	3,800 (2,750, 5,020)	5,505 (3,810, 7,255)	−5.730	<0.001
① Intraoperative blood loss (mL)	400 (300, 750)	800 (600, 1200)	−7.350	<0.001
② Intraoperative blood transfusion			16.187	<0.001
NO	101 (43.7)	12 (17.1)		
Yes	130 (56.3)	58 (82.9)		
① APTT (seconds)	29.80 (26.90, 33.70)	32.90 (29.05, 40.62)	−4.134	<0.001
① PT (seconds)	14.30 (13.10, 15.70)	15.45 (14.00, 17.28)	−3.639	<0.001
④ Albumin (g/L)	30.88 ± 6.00	25.11 ± 6.90	6.311	<0.001
① Hemoglobin (g/L)	101.00 (87.50, 112.00)	92.50 (75.50, 104.00)	2.887	0.004
① Fibrinogen (g/L)	2.44 (1.88, 2.91)	1.77 (1.36, 2.21)	5.448	<0.001
② Postoperative use of sedative drugs			7.759	0.005
No	183 (79.2)	44 (62.9)		
Yes	48 (20.8)	26 (37.1)		
② Postoperative mechanical ventilation			9.775	0.002
No	179 (77.5)	41 (58.6)		
Yes	52 (22.5)	29 (41.4)		

**Notes.**

① Indicates variables described in Median (Q1, Q3) form, and the rank-sum test was used for analysis.

② Representation of this variable is described in the form of n (%), and the chi-square test was used for analysis.

③ Representation of this variable is described in the form of n (%), and the Fisher’s precision probability test was used for analysis.

④ Indicates variables described in mean ± standard deviation form and independent samples *t*-test for analysis of variance.

NRS 2002 Nutritional Risk Screening 2002 ASA American society of Aneshesiologists physical status classification system APTT activated partial thromboplastin time PT prothrombin time

### Multivariable logistic regression analysis of hypovolemic shock in training group

**Figure 1:**
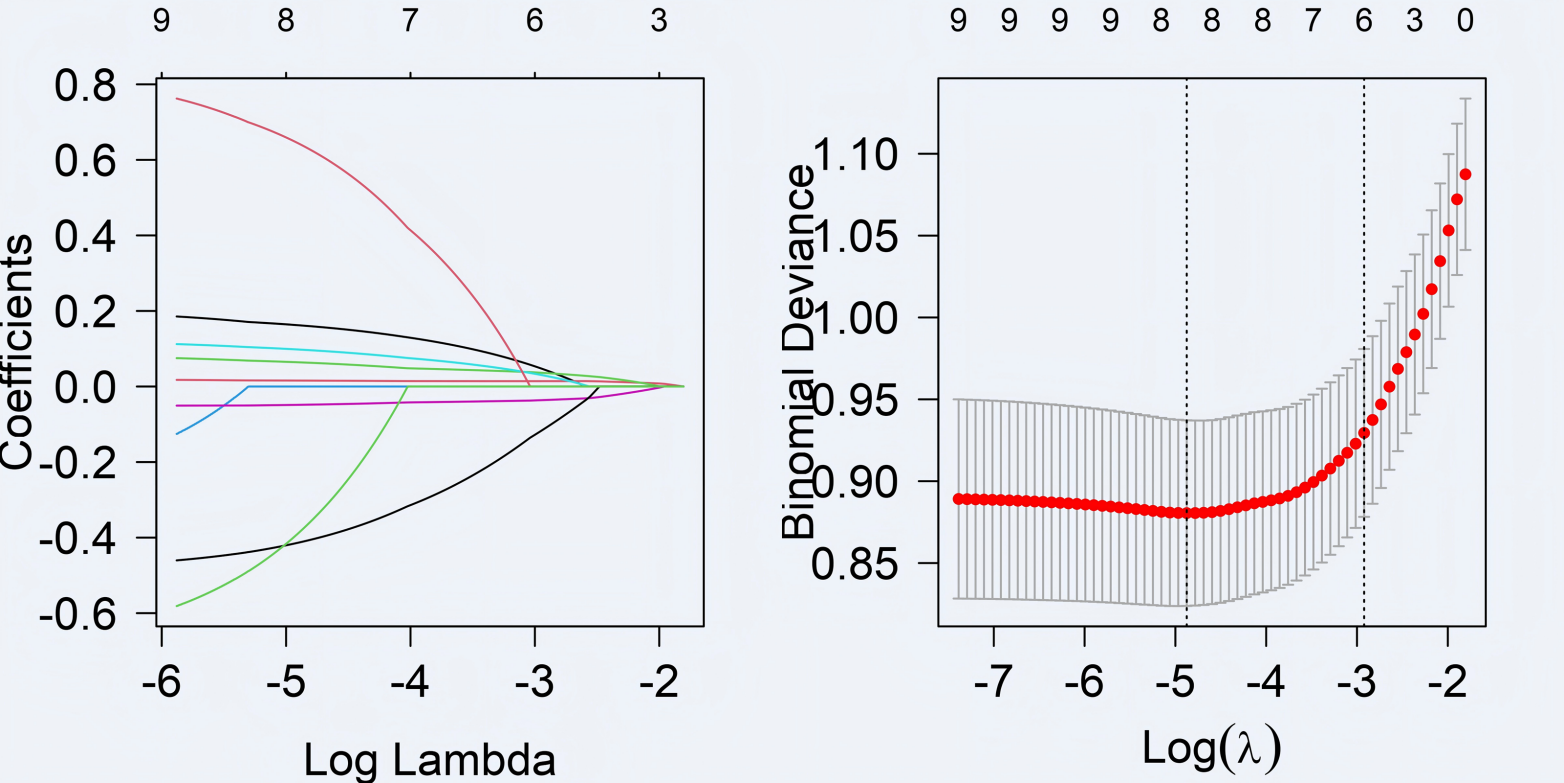
The influencing factors were screened by LASSO logistic regression model. Log Lambda (*X*-axis): The logarithm of the regularization parameter *λ*. A smaller value indicates a lower *λ*, resulting in weaker penalty on model coefficients. A larger value means a higher *λ*, leading to stronger penalty and a simpler model (more coefficients are compressed to zero). Coefficients (*Y*-axis): These represent the estimated coefficients (or weights) of each independent variable in the model. Each colored curve shows how the coefficient of a variable changes with *λ*. When *λ* is small, coefficients can vary freely, indicating a more complex model. As *λ* increases, coefficients gradually compress toward zero, with less significant variables becoming zero, thereby achieving variable screening. Log(*λ*): The logarithm of the regularization parameter *λ*. Binomial deviance: This metric evaluates model fit quality, with lower values indicating better performance. The red curve displays average binomial deviations across different *λ* values, while gray regions represent confidence intervals. Two vertical dashed lines in the graph indicate the optimal *λ* values selected based on specific criteria.

Based on the results of univariable logistic regression analysis, the LASSO regression algorithm was used to select significant features (coefficients were non-zero). In addition, 10-fold cross-validation was applied to determine the optimal parameter configuration. Then, coefficients were determined based on the lambda value (min) corresponding to the minimum distance deviation, and variables with non-zero coefficients were screened out (Fig. 1). Based on the selected influencing factors, multivariate logistic regression analysis (step-by-step, two-way) was performed, and the results showed that operation duration, intraoperative blood loss, immediate postoperative albumin and fibrinogen, and postoperative use of sedative drugs were the influencing factors of hypovolemic shock (*P* < 0.05, Table 2).

**Table 2:** Multivariate logistic regression analysis of hypovolemic shock in training group.

**Variable**	*β*	**SE**	**Z**	**OR (95% CI)**	***P***
Operation duration	0.242	0.099	2.448	1.273 (1.052, 1.552)	0.014
Intraoperative blood loss	0.097	0.041	2.384	1.102 (1.020, 1.196)	0.017
PT	0.125	0.070	1.791	1.133 (1.005, 1.313)	0.073
Albumin	−0.067	0.031	−2.174	0.935 (0.879, 0.993)	0.030
Fibrinogen	−0.501	0.239	−2.097	0.606 (0.371, 0.948)	0.036
Postoperative use of sedative drugs					
No	0.000			reference	
Yes	0.810	0.358	2.264	2.248 (1.109, 4.538)	0.024
Postoperative mechanical ventilation					
No	0.000			reference	
Yes	−0.662	0.409	−1.617	0.516 (0.224, 1.122)	0.106

**Notes.**

PT prothrombin time

### Construction of predictive models

**Figure 2:**
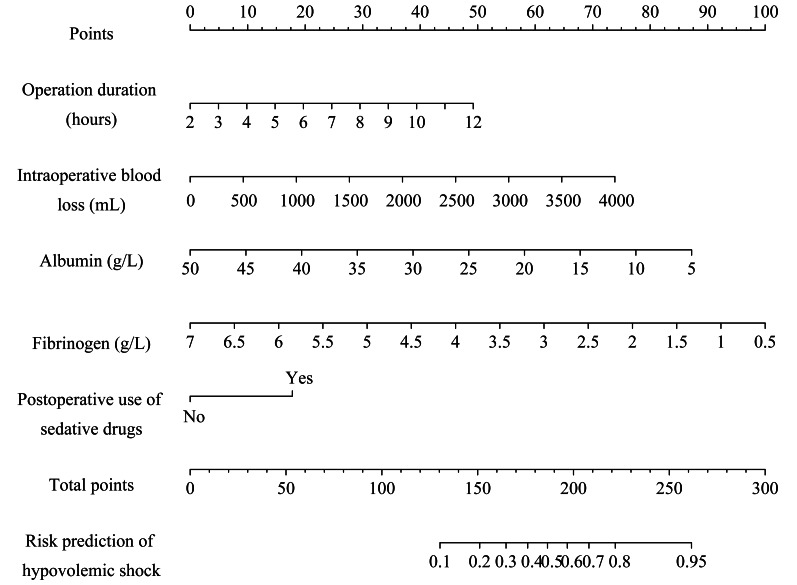
The nomogram of a risk prediction model for hypovolemic shock in ovarian cancer patients after cytoreductive surgery.

Based on the results of multivariate Logistic regression analysis, significant variables (*P* < 0.05) were used as independent predictors, including operation duration, intraoperative blood loss, immediate postoperative albumin and fibrinogen, and postoperative use of sedative drugs. Subsequently, we established a nomogram using these predictors (Fig. 2). The first line in the figure represents the score corresponding to a single variable. A vertical line was drawn up for each variable to obtain the corresponding score value, and the score corresponding to each variable was added together to obtain the total score. The corresponding score was found in the second-to-last line (total score), and then the vertical line was drawn down to find the corresponding point in the last line to obtain the risk probability of hypovolemic shock. For example, the operation duration of a patient was 8 *h* = 29 points, the intraoperative blood loss was 3,000 ml = 55 points, albumin was 20 *g*/*L*  = 57.5 points, fibrinogen was 2.5 *g*/*L* = 68.5 points, and sedative drugs was used after the operation = 17.5 points. The total score was 29 + 55 + 57.5 + 68.5 + 17.5 = 227.5 points. According to the nomogram, the risk probability of this patient developing hypovolemic shock after the operation is approximately 80%.

### Validation of risk prediction models: ROC curve

**Figure 3:**
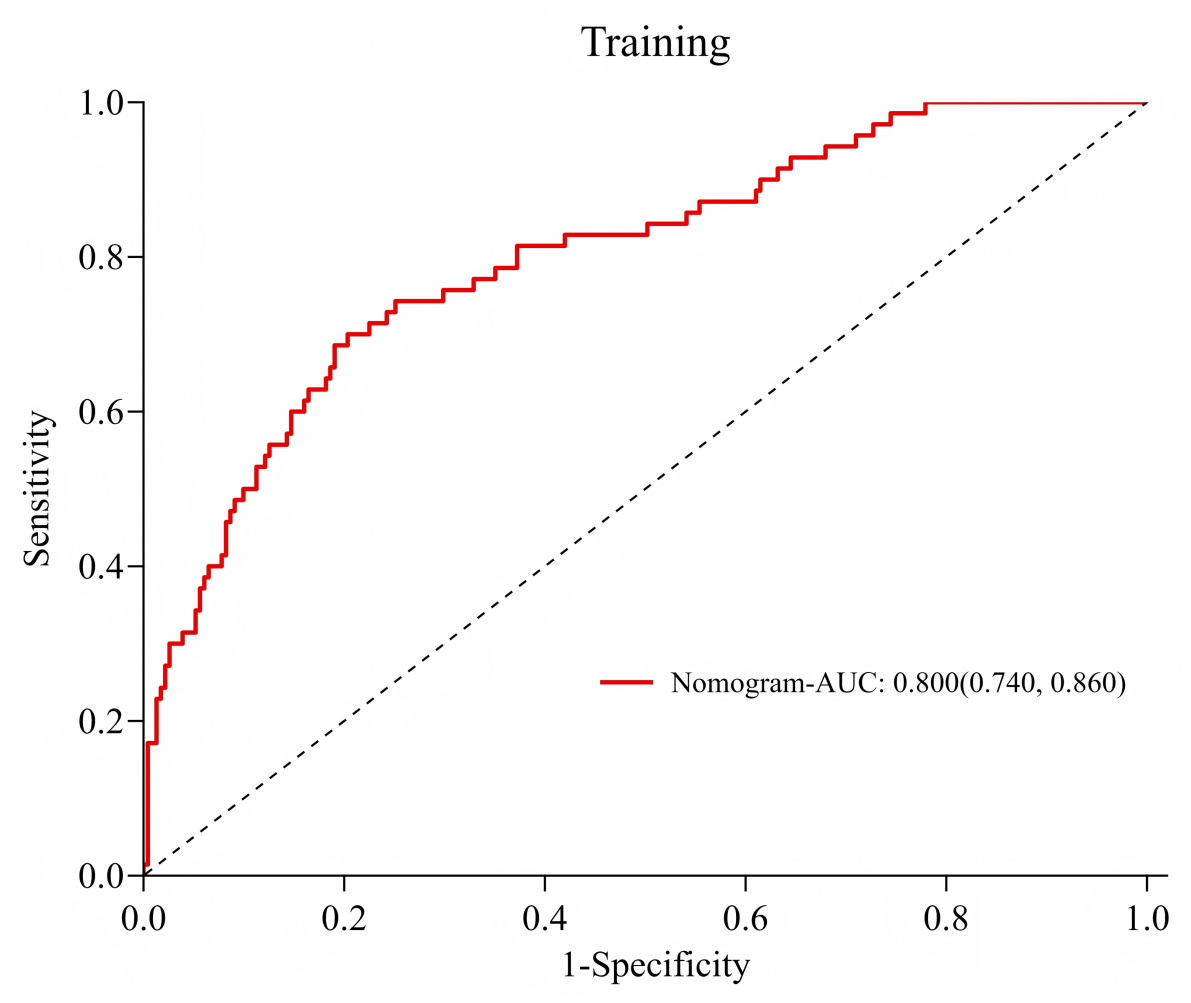
ROC curve of the training group. 1-Specificity (*X*-axis): This metric measures the percentage of cases where the model incorrectly classifies samples that are actually negative as positive. Sensitivity (*Y*-axis): This metric evaluates the model’s ability to accurately identify all samples that are actually positive.

**Figure 4:**
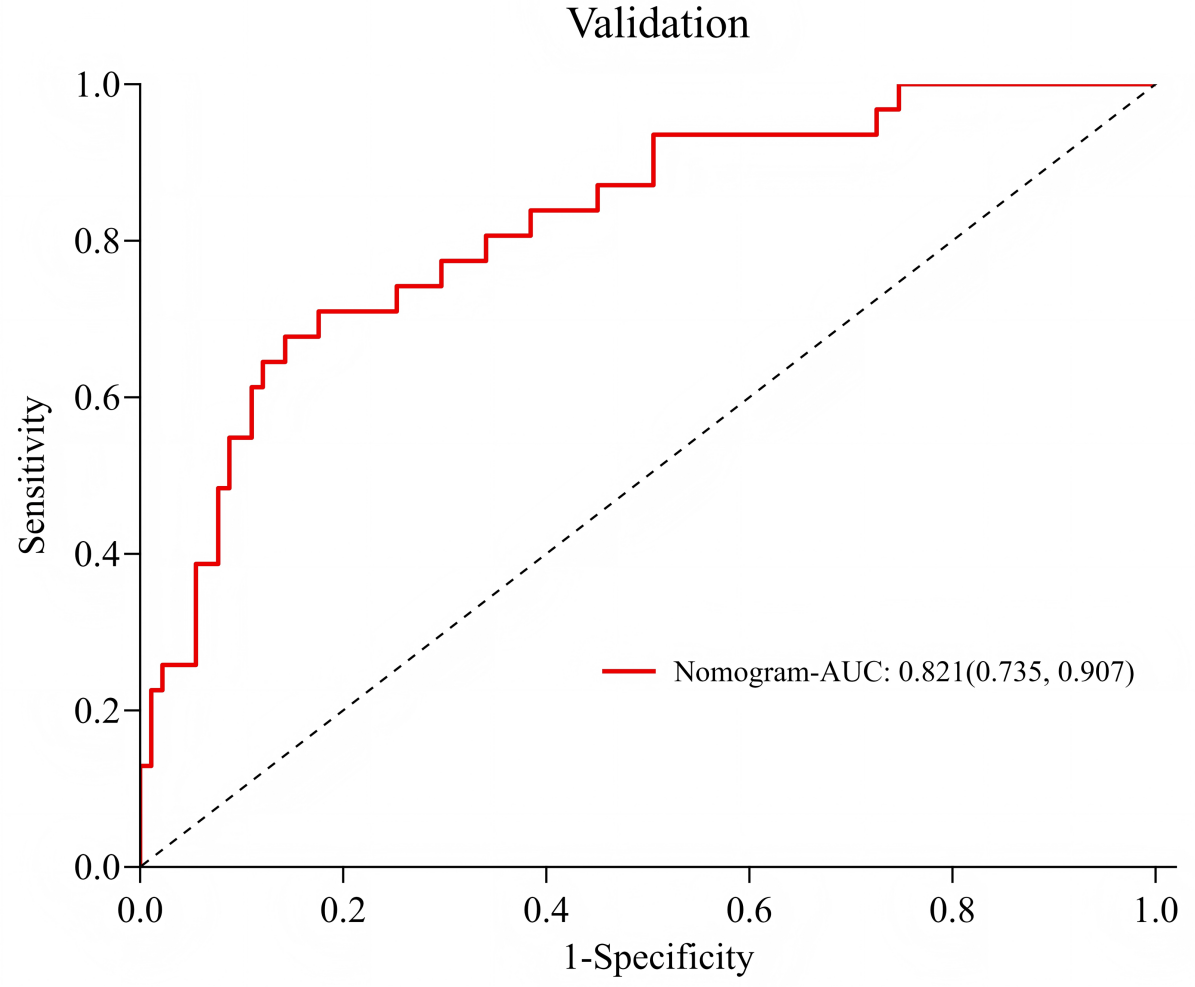
ROC curve of the validation group. Consistent with Fig. 3.

Among 122 patients in the validation group, 31 cases (25.4%) developed hypovolemic shock. ROC curve analysis results showed that the AUC of the training group and the validation group were 0.800 (95% CI [0.740–0.860]) and 0.821 (95% CI [0.735–0.907], Figs. 3–4), respectively, and the accuracy was 77.4%, indicating that the model had good prediction efficiency. The sensitivity and specificity of the training group were 70.0% and 79.7%, and the sensitivity and specificity of the validation group were 67.7% and 85.7%, respectively. The accuracy rate was 81.1%, indicating that the model had strong recognition ability and high accuracy.

### Validation of risk prediction models: calibration curve

**Figure 5:**
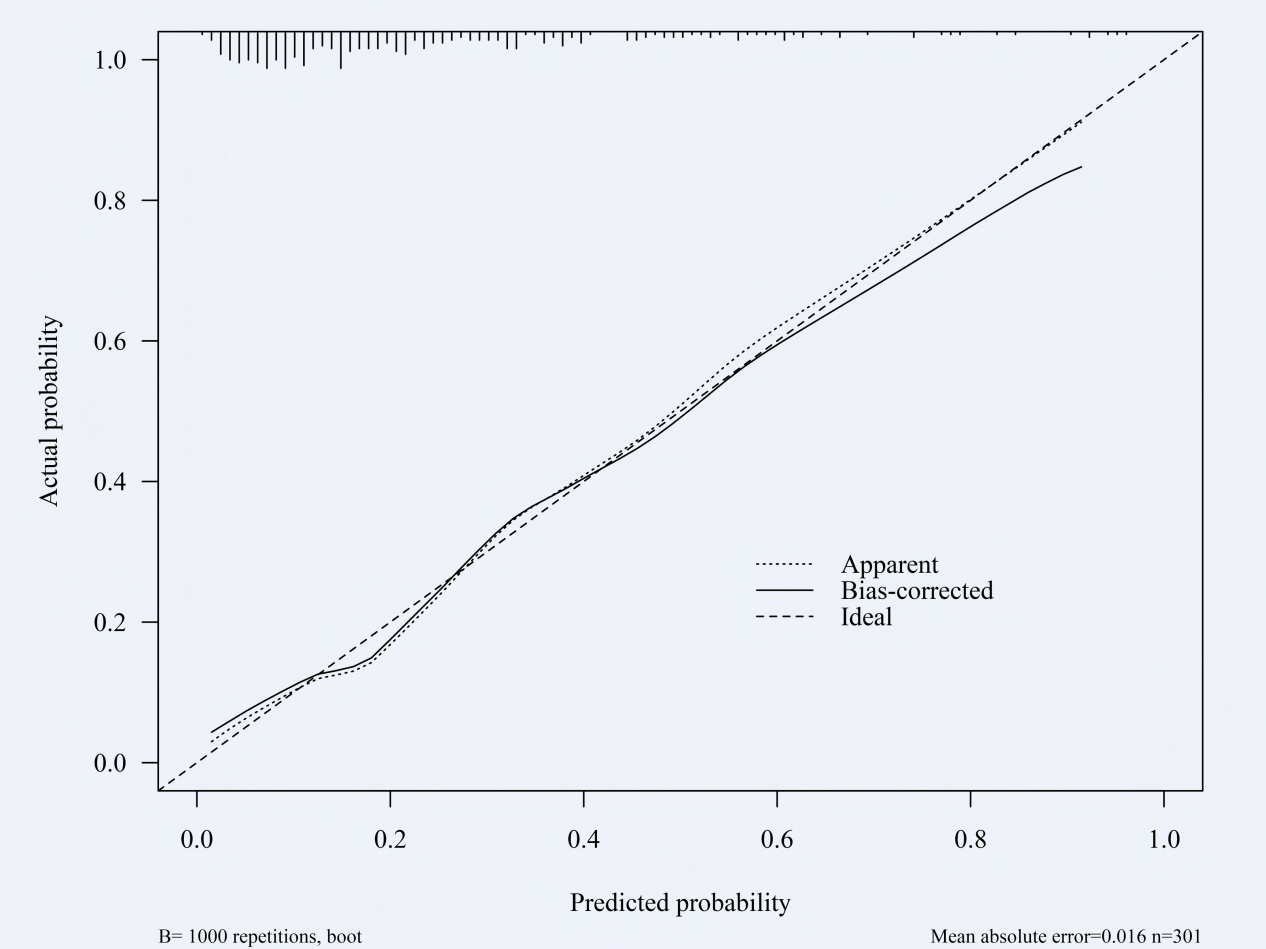
Calibration curve of the training group. Predicted probability (*X*-axis): The probability of hypovolemic shock predicted by the model. Actual probability (*Y*-axis): The actual frequency of an event observed within the corresponding predicted probability group. Ideal: This line represents the perfect calibration state, where the predicted probability matches the actual probability exactly. Apparent: This indicates the calibration performance of the model on the training dataset. Bias-corrected: This estimate is derived by applying statistical methods like Bootstrap resampling to correct deviations in the performance curve. It more accurately reflects the model’s true calibration performance on new, unknown data, making it a key metric for evaluating model calibration.

**Figure 6:**
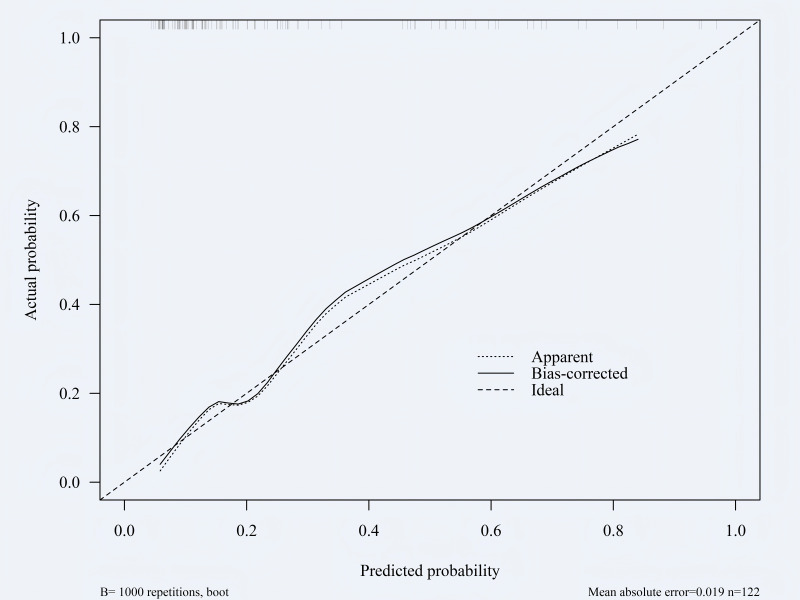
Calibration curve of the validation group. Consistent with Fig. 5.

The constructed nomogram was verified by bootstrap sampling (internal bootstrap sampling verification 1,000 times), and calibration curves were drawn (Figs. 5–6). In the training group, the model prediction probability was basically consistent with the actual incidence rate, with a mean absolute error of 0.016; in the validation group, the model prediction probability was basically consistent with the actual incidence rate, with an average absolute difference of 0.019, indicating that the model was accurate. The Hosmer-Leme-show test results of the training group showed *X*^2^ = 5.327, *P* = 0.722, and the Hosmer-Leme-show test results of the validation group showed *X*^2^ = 6.740, *P* = 0.565, indicating a good fit of the model.

### Validation of risk prediction models: DCA curve

**Figure 7:**
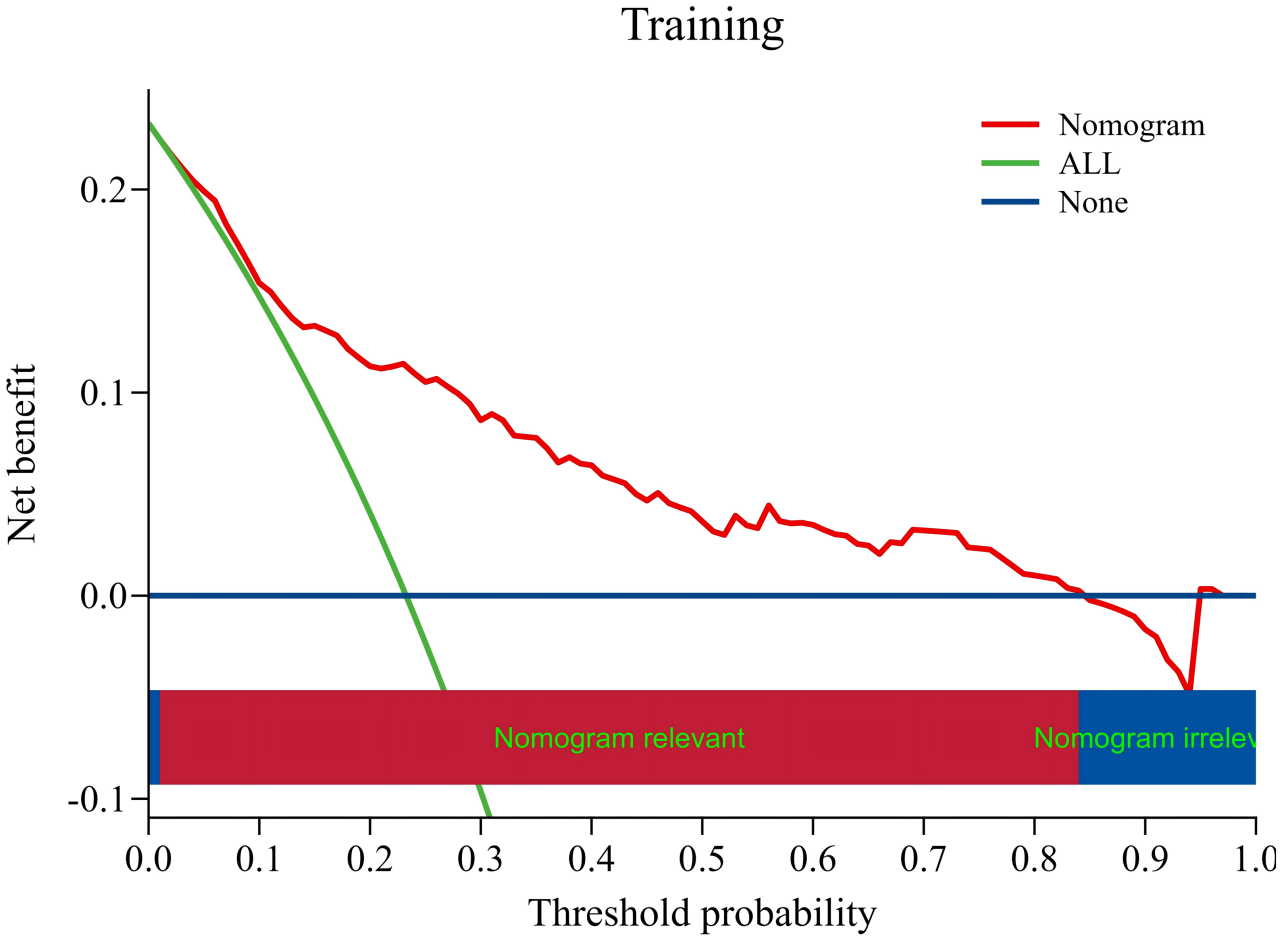
DCA curve of the training group. Threshold probability (*X*-axis): The probability threshold set by the decision-maker in clinical practice. This threshold is a prior probability indicating that when the predicted probability of an event exceeds this critical point, the decision-maker considers the benefits of intervention to outweigh its risks and costs. Net benefit (*Y*-axis): The standardized net gain achieved by a specific prediction model or strategy compared to the baseline strategy. This metric evaluates the utility of beneficial interventions (true positives) and harmful interventions (false positives) on a unified scale.

**Figure 8:**
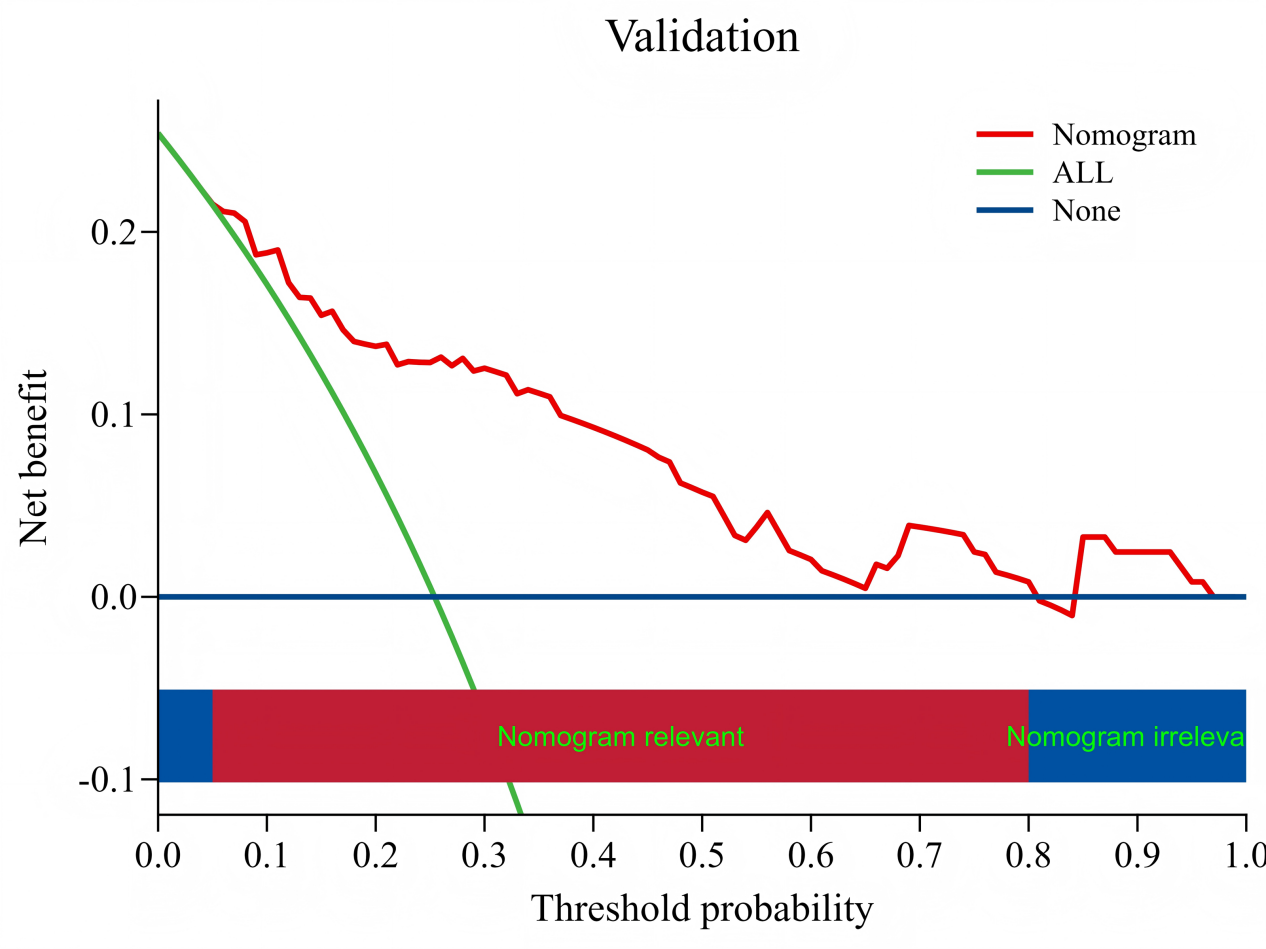
DCA curve of the validation group. Consistent with Fig. 7.

In the DCA curve, the vertical coordinate represents the net benefit. For the model, what is calculated is the benefit of the patient from the model. The benefit is the true positive rate of the model, because this part of the patients is correctly predicted and can benefit from clinical treatment. The cost was a false positive rate, and these patients received unnecessary clinical treatment because of the model. Therefore, the greater the net benefit, the greater the application value of the model in practice. The results of the DCA curve showed that the training group and the validation group had a net benefit within the probability range of risk threshold value of 0.01 to 0.84 and 0.04 to 0.80, respectively, and the net benefit of clinical intervention based on the constructed model was higher than that of intervention for all patients and no intervention for all patients (Figs. 7–8).

## Discussion

The prediction model constructed in this study not only includes the intraoperative and postoperative conditions of the patients, but also includes laboratory test indicators that can more accurately reflect the postoperative circulation and blood volume of the patients, which can realize the dynamic assessment and prediction of the risk of hypovolemic shock after cytoreductive surgery. In this study, the ROC curve, calibration curve, and DCA curve showed that the model had good clinical diagnostic efficiency. The influencing factors in the model are easily obtained from the patient’s medical record system, and medical staff may be able to evaluate the risk of hypovolemic shock after cytoreductive surgery in ovarian cancer patients according to the predictive factors, and actively take corresponding measures. This study provides necessary guidance for optimizing clinical decision-making and improving patient prognosis.

This study demonstrated that the incidence of hypovolemic shock after cytoreductive surgery in patients with ovarian cancer was 23%, which was significantly higher and primarily associated with the characteristics of the enrolled population, surgical approach, and the rigor of diagnostic and monitoring protocols. The specific analysis is as follows: First, surgical trauma was the core etiological factor. All enrolled patients underwent open tumor cytoreductive surgery, which required extensive exposure of the surgical field and extensive peritoneal dissection to achieve complete tumor resection. The intraoperative blood loss and fluid loss were significantly higher than those in non-open surgeries, making postoperative hypovolemic shock more likely due to insufficient blood volume. Second, the high-risk attributes of the enrolled population increased the risk of shock. All study subjects required intensive care unit (ICU) transfer postoperatively, and most of these patients had advanced tumor stages, significant intraoperative blood loss, and some had underlying diseases or preoperative anemia, which reduced their tolerance to blood volume fluctuations. Consequently, the incidence of shock may be higher than that in the general postoperative population. Additionally, the strict diagnostic criteria and the high-sensitivity monitoring in the ICU ensured accurate identification of all cases of hypovolemic shock. In the ICU, healthcare professionals can promptly identify the early and transient signs of shock, and most cases received vasopressors to maintain hemodynamic stability after diagnosis. A minority of patients who did not receive vasopressors achieved rapid shock correction through timely and adequate fluid resuscitation. These cases, which might be overlooked in general wards, were included in the statistics, objectively increasing the incidence rate. In summary, the high trauma of open surgery, the high-risk nature of the enrolled population, and the rigor of diagnostic and monitoring protocols collectively contributed to the higher incidence of postoperative hypovolemic shock in this study. This data objectively reflects the true complication status of high-risk patients and can provide references for optimizing perioperative management and reducing the risk of shock.

The duration of operation and the amount of blood loss are important factors affecting the prognosis and survival of patients undergoing major surgery (Fagotti et al., 2020; Rekman et al., 2017). The results of this study showed that patients with longer operation time and more intraoperative blood loss were more likely to develop hypovolemic shock. The reason may be that most ovarian cancer patients are accompanied by multiple pelvic and abdominal metastases at diagnosis. In order to achieve the surgical effect without gross visual residual, the peritoneal exfoliation area is extensive (Angeles et al., 2019; Angeles et al., 2020), and multiple organs such as the colon, diaphragm, spleen, pancreas, and liver need to be resected or partially resected (Tse et al., 2025), resulting in prolonged operation time and increased intraoperative blood loss. If the lost blood volume cannot be effectively replaced and the blood volume level maintained, the risk of postoperative hypovolemic shock will be further increased. Therefore, the intraoperative and postoperative risk assessment should be strengthened for patients with long operation time and large amount of blood loss, and the operation time should be shortened by optimizing the technology and process on the premise of ensuring the quality of the operation. Simultaneously, continuous monitoring of patients’ circulatory status and blood volume is essential, alongside close surveillance of laboratory parameters such as complete blood count, biochemical markers, and other relevant indicators. Prompt fluid replacement should be administered to compensate for intraoperative losses, and blood transfusions should be initiated when indicated to maintain adequate tissue perfusion. These measures aim to prevent hypovolemic shock resulting from excessive blood loss. It has been observed that adherence to the enhanced recovery after surgery (ERAS) protocol results in a shorter duration of operation relative to conventional care (Roy et al., 2025). This highlights the role of healthcare professionals in employing multimodal, multidisciplinary perioperative methods to expedite functional recovery, shorten the duration of the operation, and thereby ameliorate both intraoperative and postoperative outcomes.

Previous studies have shown that the decrease of perioperative albumin level is not only associated with adverse outcomes after various surgical operations (Wilson et al., 2019; Joliat et al., 2022), but also an independent predictor of severe postoperative complications of ovarian cancer (Ataseven et al., 2015). During cytoreductive surgery, plasma albumin levels usually decrease due to traumatic inflammation and increased capillary permeability (Amouzandeh et al., 2018). In addition, one study showed that ascites occurred in more than one-third of ovarian cancer patients (Kipps & Tan, 2013). Due to the high content of albumin in ascites (Ohashi et al., 2019), intraoperative ascites loss may result in a decrease in albumin levels. If it is not corrected in time, it will further lead to tissue hypoperfusion and cause hypovolemic shock. Nutritional support and preoperative exercise training facilitate the storage of protein in the body as muscle protein, thereby avoiding severe catabolism and the subsequent loss of body protein, strength, and function. It has been documented that implementing prehabilitation strategies based on exercise and nutritional support before surgery can increase patients’ postoperative albumin levels and improve their nutritional status (Xiao et al., 2024). Therefore, clinical healthcare providers should place emphasis on preoperative prehabilitation strategies, enhance preoperative nutritional support and exercise training, improve cardiopulmonary function and energy reserves, minimize surgical stress responses to the greatest extent, and contribute to the maintenance of homeostasis. For patients with low albumin levels after surgery, accurate target-oriented fluid management can be used to maintain colloid osmotic pressure (Gomez-Izquierdo et al., 2017), correct perioperative hypovolemia, avoid tissue hypoxia, and ensure organ function (Wiedermann, 2020).

The results of this study showed that patients with lower fibrinogen were more likely to develop hypovolemic shock after surgery. Plasma fibrinogen, a precursor of fibrin, plays a crucial role in hemostasis by promoting clot formation and platelet aggregation through binding to platelet glycoprotein IIb/III receptors (Huang et al., 2023; Hayakawa et al., 2011). One study showed that the fibrinogen plasma concentration during surgery determined the amount of blood loss and the need for transfusion (Skrtic et al., 2017). Cytoreductive surgery has a wide range of operations, complicated surgical methods, and serious vascular damage, resulting in a large amount of blood loss. It is suggested that medical staff should pay attention to the fibrinogen indexes of patients, identify and give symptomatic treatment early, and effectively prevent excessive blood loss caused by reduced fibrinogen levels in patients (Grottke et al., 2019).

The results of this study show that postoperative use of sedative drugs may increase the risk of hypovolemic shock, which may be due to the respiratory and circulatory inhibition effect of sedative drugs, resulting in hemodynamic instability, decreased blood pressure and bradycardia (Tarwade, 2022; Chen et al., 2021; Ho, Chang & Lu, 2024). In this study, the types of sedative drugs used by the research subjects after surgery mainly included benzodiazepines (midazolam and remimazolam), propofol and dexmedetomidine. Remimazolam, as a new type of benzodiazepine drug, has the characteristics of rapid onset, quick recovery and mild hemodynamic side effects. Compared with midazolam, remimazolam has a shorter duration of effect and can better regulate the sedation level. Compared with propofol, remimazolam causes less vascular pain and less blood pressure drop (Morimoto, 2022). The intraoperative hypotension rate was lower in the remimazolam (22%) than in the propofol (49%) group in a clinical trial that investigated patients who received general anesthetics (Doi et al., 2020). A systematic review and meta-analysis comparing different sedatives in critically ill adults with mechanical ventilation indicated that, compared with benzodiazepines and propofol, the use of dexmedetomidine increased the risk of bradycardia (risk ratio (RR) 2.39, 95% CI [1.82–3.13]) and hypotension (RR 1.32, 95% CI [1.07–1.63]) (Lewis et al., 2022). It is suggested that the medical staff should not only ensure the depth of sedation required by the patients, but also consider whether the sedative drugs meet the characteristics of no accumulation in the body, little inhibition of respiratory circulation and predictable dose–effect. In addition, it is worth noting that hypotension caused by sedative use may affect the differentiation and diagnosis of postoperative hypovolemic shock in patients, so hypotension cannot be used as the only condition in the identification of hypovolemic shock.

### Limitations

First of all, this study is retrospective and relies on existing data, and its accuracy, completeness and consistency may have problems, which may affect the reliability of the study results. Second, the subjects of this study were patients who underwent surgical evaluation and were deemed by surgeons to require transfer to the ICU for transitional care postoperatively. These patients generally exhibit characteristics such as extensive surgical resection, significant intraoperative blood loss, and relatively complex conditions, which differ significantly from the general population cohort that was directly returned to general wards postoperatively. Consequently, the incidence of hypovolemic shock in this subgroup cannot be directly compared. Through a review of existing international literature on cytoreductive surgery and hyperthermic intraperitoneal peroperative chemotherapy for ovarian cancer, data on the incidence of hypovolemic shock in high-risk subgroups of patients transferred to the ICU postoperatively remain relatively limited, making precise cross-sectional comparisons currently unfeasible. Third, this study did not conduct subgroup analyses on factors such as the type, duration, and dosage of sedatives used postoperatively. Future research could further investigate the impact of these different subgroups on the occurrence of postoperative hypovolemic shock to enhance relevant clinical evidence. In addition, this study is based on single-center data, with a limited sample size and a lack of external validation. Therefore, caution should be exercised when interpreting our results. In future studies, it is necessary to include the entire population that directly returns to general wards postoperatively to compare the incidence of hypovolemic shock among patients with different characteristics. Meanwhile, multi-center and large-sample studies need to be conducted to further evaluate the robustness and clinical applicability of this model.

## Conclusions

This study showed that operation duration, intraoperative blood loss, immediate postoperative albumin and fibrinogen, and postoperative sedative use were independent influencing factors for hypovolemic shock after cytoreductive surgery in ovarian cancer patients. The risk prediction model built based on these influencing factors had good differentiation and accuracy. It can provide scientific basis for clinical staff to predict hypovolemic shock risk and formulate intervention strategies as soon as possible.

##  Supplemental Information

10.7717/peerj.20976/supp-1 Supplemental Information 1 Raw data

## Additional Information and Declarations

### Data Availability

The following information was supplied regarding data availability: The raw data are available in the Supplemental File.
